# Efficacy of Caregivers Training in Rehabilitation of Stroke Survivors in Bangladesh: A Quasi-experimental Study

**DOI:** 10.7759/cureus.33812

**Published:** 2023-01-16

**Authors:** Mahbubul Islam, Nuruzzaman Khandaker, Md. Shahidur Rahman, Anjuman Sultana, Khadiza Yasmin, Puza Das Dewan, Md. Hasibul Islam, Kamrul Hasan, Redoy Ranjan

**Affiliations:** 1 Physical Medicine and Rehabilitation, Directorate General of Health Services (DGHS), Dhaka, BGD; 2 Physical Medicine and Rehabilitation, Bangabandhu Sheikh Mujib Medical University (BSMMU), Dhaka, BGD; 3 Gynecologic Oncology, Directorate General of Health Services (DGHS), Dhaka, BGD; 4 Anatomy, Bangabandhu Sheikh Mujib Medical University (BSMMU), Dhaka, BGD; 5 Cardiovascular Surgery, National Institute of Cardiovascular Diseases, Dhaka, BGD; 6 Surgical Science, The University of Edinburgh, Edinburgh, GBR; 7 Institute of Cardiovascular Research, Royal Holloway University of London, London, GBR; 8 Cardiac Surgery, Bangabandhu Sheikh Mujib Medical University (BSMMU), Dhaka, BGD

**Keywords:** physiotherapy education, functional independence measure (fim), the london stroke carers training course, caregivers training in rehabilitation, stroke

## Abstract

Background: A proper rehabilitation program may prevent post-stroke neurological, structural, and functional disabilities. We aimed to evaluate the efficacy of caregiver training in the rehabilitation of stroke survivors and compare rehabilitation interventions done by the therapist.

Methods: This quasi-experimental study was conducted among 67 stroke survivors divided into group A (home-based exercise by family caregivers; n=33) and group B (hospital-based supervised exercise by a physiotherapist; n=34). Family caregivers were trained according to “The London Stroke Carers Training Course.” The functional independence measure (FIM) evaluated all patients after three months of physiotherapy.

Results: The mean age of the participants in group A and group B were 56.85 ± 11.49 and 58.65 ± 16.92 years, respectively, where most of the patients in both groups were male. In group A, 17 (51.5%) participants had left-sided involvement, while in group B, 17 (50.0%) participants had left-sided involvement. There was no significant statistical difference in FIM between groups A and B at baseline (p=0.532). At three months, the mean FIM of the participants in group A (98.54 ± 11.85) was significantly higher than in group B (89.85 ± 8.15) (p=0.001). A quasi-significant difference was observed between the right (18.41 ± 9.37) and the left (23.42 ± 11.68) hemisphere involvement regarding mean improvement of FIM (p=0.057).

Conclusion: Therapeutic approach provided by trained caregivers was found to be more effective and efficient than that done by a physiotherapist.

## Introduction

Strokes cause motor, sensory, perceptual, and cognitive impairments. These deficits can lead to disability and hamper functional capability [1]. Ischemic causes of stroke are related to thrombotic, embolic, or hemodynamic factors, while hemorrhagic strokes are either intracerebral or subarachnoid in location [2]. In 2017, 104.2 million people worldwide had a stroke, with 82.4 million having an ischemic stroke, 17.9 million having an intracerebral hemorrhage, and 9.3 million having subarachnoid bleeding. The most remarkable prevalence rate of ischemic stroke was seen in Eastern Europe and Central and East Asia, while the prevalence of intracerebral hemorrhage was high in East and Central Asia [2-3]. Stroke is the third leading cause of mortality in Bangladesh. The reported prevalence of stroke in Bangladesh is 0.3%, where the ratio of male: female patients is 3.44: 2.414. According to the demographic statistics, 54.0% and 46.0% of the population resided in urban and rural regions, respectively, while 47.0% and 53.0% were from the low- and middle-income categories [4]. 

After a stroke, patients may experience a variety of consequences, especially behavioral changes, aphasia, and disability in doing daily activities [5] that can be prevented or efficiently addressed by a proper rehabilitation program, and exercise has been shown to enhance mobility and balance in stroke patients in many studies [5-6]. The result of the controlled trial in Bradford shows that at-home physiotherapy for stroke patients is more effective than rehabilitation at a day in hospital in reducing disability [7]. Patients managed at home had lower depression scores, fewer complications, and were more likely to remain at home [8]. The London stroke carers training course is a systematic training program designed to meet the requirements of inpatients who are expected to return home following a stroke [8-9]. Diet, nutrition and feeding, continence care, bed placement, activities of daily living (ADL), speech therapy, swallow therapy, and physiotherapy were among the items demonstrated in the London Stroke Carers Training Course [7-10]. 

Stroke rehabilitation in Bangladesh is unaddressed primarily due to a lack of resources, and after receiving acute care in a hospital, most stroke survivor patients return to their distant communities with partial recovery and profound disability [11]. Physiotherapy and nursing care are rarely taught or briefed to caregivers who manage patients at home, and majority of individuals return for their next appointment with several issues [11]. Till now, in Bangladesh, there is a scarcity of research on this topic, so this work was done to evaluate the efficacy of caregiver training in the rehabilitation of stroke survivors and to compare it with the rehabilitation intervention done by a physiotherapist. This study aimed to assess the effectiveness of home-based rehabilitation provided by caregivers and rehabilitation given by physiotherapists to improve the disability following acute stroke.

## Materials and methods

This quasi-experimental study was conducted in the department of physical medicine & rehabilitation, Bangabandhu Sheikh Mujib Medical University (BSMMU), Bangladesh, between March 2020 and September 2021. A total of seventy-three patients with four weeks of stroke with hemiparesis or hemiplegia attending the outpatient and inpatient department of physical medicine and rehabilitation and inpatient department of neurology, BSMMU, were purposively selected for the study. However, six patients lost follow-up, apparently without clinical reasons, and patients were classified into group A (n=33) and group B (n=34). Study group A included patients with home-based therapeutic exercises, which family caregivers did according to muscle power; group B included patients treated with a hospital-based supervised exercise program by physiotherapists in a hospital or at home five days a week. Patient's allocation into group A was based on their home location (distance to our university hospital) and financial status. We emphasized the caregiver training program for the group A population because patients are required to pay themselves for every session with the physiotherapist, which is comparatively higher than caregivers, and it is not convenient to travel from a remote area or district town to our University hospital regularly. However, in this study, no randomization was done, and patients were allowed to participate in any rehabilitation program. Seriously ill patients like unconscious patients, previous history of recent myocardial infarction (MI), unstable angina, bronchial asthma, dyslipidemia, uncontrolled diabetes mellitus, uncontrolled hypertension and patients with significant cognitive deficits were excluded from the study. Ethical clearance was obtained from the institutional review board (IRB reference BSMMU/2020/4763) of BSMMU and informed written consent was taken from the patients or next of kin. According to obtained history, the demographic profile of the patients was recorded. Functional assessment of the patients was done using the Bengali version of the functional independence measure (FIM) score as it is easy and convenient to make them understand the scoring system, explained details elsewhere [12].

Caregiver training program

The caregiver's training was given according to "The London Stroke Carers Training Course" [9], and the same caregiver was selected for the same patient who could understand the training procedure. Moreover, a physiotherapist trained family caregivers under the supervision of the researcher at the first visit. This training has mandatory elements like health education, lifestyle modification, and secondary preventions like control of hypertension (HTN), use of aspirin/warfarin, avoidance of smoking, exercise etc. Furthermore, appropriate elements of the caregiver training program were medication compliant, post-discharge arrangements and adapting the skills about their home environment (use of high commode or chair commode, modification of ramps for wheelchair use, change of bathing elements, rearranging clothing before and after bathing) were touched. Some relevant aspects were also connected, like bed positioning, speech therapy, swallow rehabilitation, bowel and bladder rehabilitation, management of pressure area, transfer techniques like bed to wheelchair or wheel to bed, transfer to toilet, use of walking aids like a cane.

Physiotherapists working in the department of physical medicine & rehabilitation, BSMMU, were selected for the study, and the same physiotherapist was enrolled for the same patient. Here exercise was also given according to the muscle power, and some essential points of rehabilitation like bed positioning, speech therapy, diet, swallow rehab, bowel and bladder management, pressure area management, and transfer technique were also touched. In both groups, exercise was done three to four times daily with five repetitions each for five days weekly. The training was dependent according to the side of the involvement. Patients of both groups were advised to follow up for three weeks to three months to see the pattern and appropriateness of training. FIM was measured at the first and last follow-up within three months. Functional improvement was assessed in the FIM score in both study groups.

Statistical analysis

The statistical analysis was conducted using SPSS (statistical package for social science) version 26 statistical software (IBM Corp., Armonk, NY). A comparison of categorical data was assessed using the Chi-square test, and associations of continuous data were evaluated using student’s t-test where p<0.05 was considered significant.

## Results

**Figure 1:**
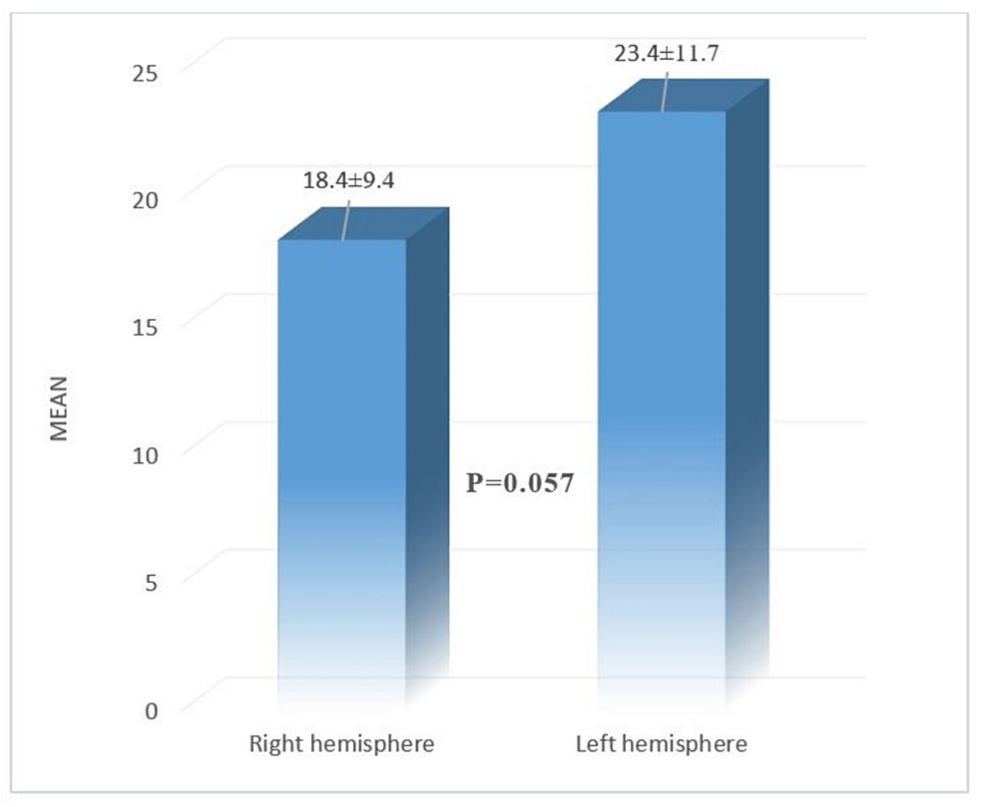
Comparison of mean improvement of FIM according to lesion location (n=67). Here, p value reached from Independent t test and p ≤ 0.05 considered as significant. FIM, functional independence measure

**Figure 2:**
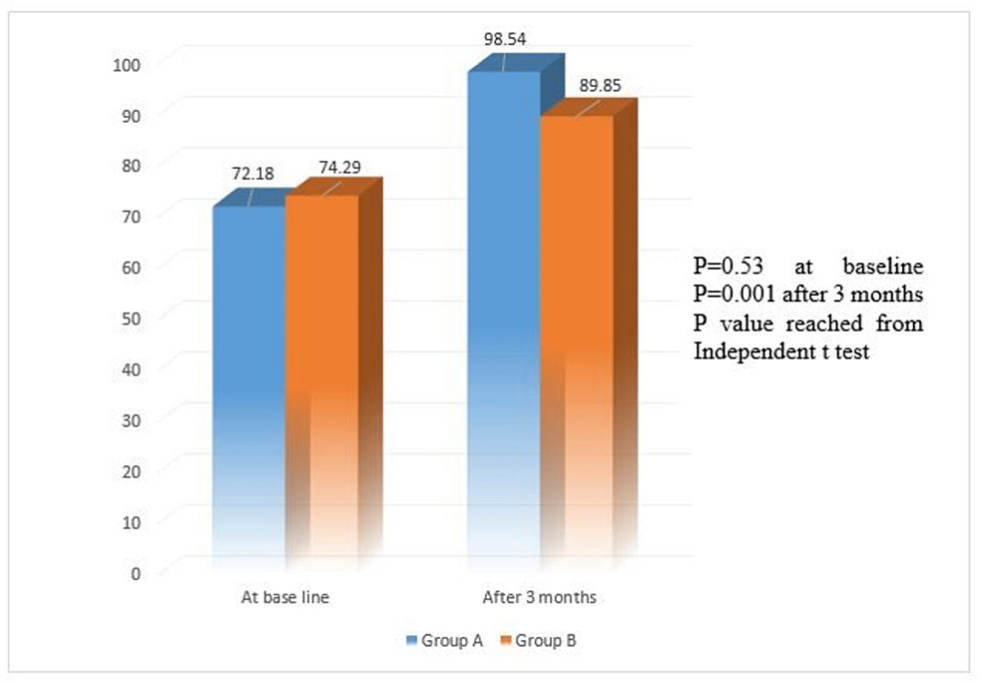
Comparison of patients by FIM (n=67). Here, p value reached from Independent t test and p ≤ 0.05 considered as significant. FIM, functional independence measure

Although 73 patients were enrolled in the study, six dropped from the study within the study period. In both groups, most patients were male (group A: 51.5% and group B 67.6%). The mean age of the patients in group A and group B was 56.85 ± 11.49 and 58.65 ± 16.92 years, respectively. No significant statistical difference was found between the groups regarding gender, age, and occupational status as p>0.05 (Table 1). No significant statistical difference was found between the groups regarding side involvement and type of stroke (p>0.05) (Table 1). At baseline in group A, the mean FIM of the patients with left hemisphere lesions was 79.94 (±13.32), which was significantly higher than the mean FIM of the patients with the right hemisphere lesion, which was 63.93 (±17.78) (p=0.006). After three months in group A, the mean FIM of the patients with left hemisphere lesions was 105.47 (±9.44), which was significantly higher than the mean FIM of the patients with the left hemisphere lesion, which was 91.19 (±9.60) (<0.001). Though at baseline, there was no significant difference in the mean FIM of the patients between two hemispheres in group B, after three months, the mean FIM of the patients with the left hemisphere lesion was 88.18 (±9.52), which was higher than the mean FIM of the patients with the left hemisphere lesion which was 91.53 (±6.34) (p=0.236) (Table 2). A quasi significant difference was observed between the right and left hemisphere involvement regarding the mean improvement of FIM as p = 0.057 (Figure 1). At baseline, the mean FIM values of the patients in group A and group B were 72.18 ± 17.40 and 74.29 ± 8.89, respectively (p = 0.532). At three months, the mean FIM of the patients in group A became significantly higher (98.54 ± 11.85) than in group B (89.85 ± 8.15) (p = 0.001) (Figure 2).

**Table 1:** Comparison of patients by socio-demographic status and clinical features (n=67). Here, p value reached from (a) Chi-square test, (b) Independent t test, and (c) Fisher's exact test, respectively and p ≤ 0.05 considered as significant. SD, standard deviation

Socio-demographic status	Group A (n=33)	Group B (n=34)	p value
Gender			
Male	17 (51.5%)	23 (67.6%)	0.178^a^
Female	16 (48.5%)	11 (32.4%)	
Age (Mean ± SD)	56.85 ± 11.49	58.65 ± 16.92	0.614^b^
Occupational status			
Home makers	13 (39.4%)	6 (17.6%)	0.152^c^
Service holder	4 (12.1%)	11 (34.2%)	
Businessman	8 (24.2%)	6 (17.6%)	
Retired person	3 (9.1%)	5 (14.7%)	
Others	5 (15.1%)	6 (17.6%)	
Monthly family income (in taka)			
Up to 10,000	16 (48.5%)	6 (17.6%)	0.007^c^
10,001-25,000	17 (51.5%)	24 (70.6%)	
Above 25,000	0 (0.0%)	4 (11.8%)	
Clinical feature			
Side of involvement			
Left	17 (51.5%)	17 (50.0%)	1.000^a^
Right	16 (48.5%)	17 (50.0%)	
Type of stroke			
Ischemic	27 (81.8%)	29 (85.3%)	0.865^b^
Hemorrhagic	5 (15.2%)	5 (14.7%)	
Both	1 (3.0%)	0 (0.0%)	

**Table 2:** Comparison of FIM according to group and lesion location (n=67). Here, p value reached from Independent t test and p ≤ 0.05 considered as significant. FIM, functional independence measure

	Group	FIM (Mean ± SD)	p value
At baseline	Group A		
	Left hemisphere	79.94 ± 13.32	0.006
	Right hemisphere	63.93 ± 17.78	
	Group B		
	Left hemisphere	76.88 ± 7.13	0.090
	Right hemisphere	71.70 ± 9.91	
At three months	Group A		
	Left hemisphere	105.47 ± 9.44	<0.001
	Right hemisphere	91.19 ± 9.60	
	Group B		
	Left hemisphere	88.18 ± 9.52	0.236
	Right hemisphere	91.53 ± 6.34	

## Discussion

The present study aimed to evaluate the efficacy of caregiver training in the rehabilitation of stroke survivors and compare it with the rehabilitation intervention given by physiotherapists. Studies conducted among the Bangladeshi stroke population also reported that most stroke patients were more than 50 years of age group [10, 13-15]. A predominance of male gender was observed in the present study, which is similar to the findings of a retrospective study conducted at the National Institute of Neuroscience (NINS) [15]. Patients of the present study were mainly housewives, service holders and businessmen, which matched with other Bangladeshi studies [14-15]. Monthly family income of patients who were given hospital-based supervised exercise was significantly higher than that of patients whose family caregivers gave home-based therapeutic exercise. In the present study, most of the patients in both groups (>81.0%) had an ischemic stroke, which is consistent with the findings of the other studies [14-18].

In stroke patients, there is a strong link between hemiparesis and functional independence [19]. From the beginning of the study, the mean FIM of the participants with the right hemisphere lesion was significantly higher than the mean FIM of the participants with the left hemisphere lesion. After three months of physiotherapy, either by caregiver or physiotherapist, the mean FIM of the participants with the right hemisphere lesion was still significantly higher than that of the participants with the left hemisphere lesion. But, the mean improvement of FIM of the participants with the left hemisphere lesion was higher than that of the participants with the right hemisphere lesion, which was statistically significant. Patients with right-sided hemiparesis are more likely than patients with left-sided hemiparesis to attain independent standing within two months after their strokes, suggesting that the side of the brain lesion may alter their total rehabilitation capacity [20]. Dhiman et al. [19] proposed that the side of hemiparesis/weakness be considered as a factor in determining functional independence and subsequent retraining for hemiparetic stroke survivors.

In both groups, there was an improvement in FIM scores after three months. But the improvement of FIM scores was higher in group A compared to group B. In a recent study, Rahman and Salek [10] observed whether caregiver training to attendees during a hospital stay can better address the stroke survivor at home and found positive effects of caregiver training at the post-acute care management of stroke survivors at home. Rahman [11] reported that caregiver home management training is helpful and improves outcomes. Also Siemonsma and coworkers [21] systematically reviewed the determinants of success of implementing home-based rehabilitation for people who experienced a recent stroke. The home environment allows for a more client-centred approach, increases patients' participation in the rehabilitation process, and calls on the problem-solving skills. Moreover, Hong et al. [22] stated that the caregiver's education program for stroke patients had a positive result on patients' functional improvement and caregiver satisfaction. Patients can gain functional improvements with additional rehabilitation treatment combined with an education program, allowing them to return to social life more quickly.

Like any study, we need to acknowledge a few limitations. This is an observational study evaluating a small sample; study results are not generalized worldwide. Caregivers were not trained in all principles of 'The London Stroke Carers Training Course,' and the study did not evaluate caregivers' satisfaction or strain. However, considering the scarcity of large, enough powered studies findings, current study outcomes are promising and rational, which could shed light on the efficacy of long-term stroke survivor management compared to the current guideline. We recommended cross-validation of our study findings with a large sample, especially enough-powered randomized controlled trials with appropriately trained caregivers.

## Conclusions

Physiotherapy given by a trained caregiver seems to be more effective and resource-efficient than rehabilitation intervention done by a therapist. In Bangladesh, in most cases, the outcomes of rehabilitation management are not satisfactory because of the inefficient and insufficient rehabilitation health workforce, and most patients cannot afford the treatment by trained healthcare workers. This study highlighted the importance of caregiver training at the resource-poor outset and reflected a promising outcome in managing stroke survivors. 
